# Iatrogenic damage to the mandibular nerves as assessed by the masseter inhibitory reflex

**DOI:** 10.1007/s10194-011-0354-0

**Published:** 2011-06-10

**Authors:** A. Biasiotta, P. Cascone, R. Cecchi, G. Cruccu, G. Iannetti, A. Mariani, A. Spota, A. Truini

**Affiliations:** 1 Department of Neurology and Psychiatry, Sapienza University, Viale Università 30, 00185 Rome, Italy; 2 Department of Maxillofacial Surgery, Sapienza University, Rome, Italy; 3 Department of Legal Medicine, Sapienza University, Rome, Italy; 4 Don Gnocchi Foundation, Rome, Italy

**Keywords:** Masseter inhibitory reflex, Exteroceptive suppression, Mandibular pain, Inferior alveolar nerve, Lingual nerve, Dental procedures, Legal litigation

## Abstract

Iatrogenic injury of the inferior alveolar or lingual nerves frequently leads to legal actions for damage and compensation for personal suffering. The masseter inhibitory reflex (MIR) is the most used neurophysiological tool for the functional assessment of the trigeminal mandibular division. Aiming at measuring the MIR sensitivity and specificity, we recorded this reflex after mental and tongue stimulations in a controlled, blinded study in 160 consecutive patients with sensory disturbances following dental procedures. The MIR latency was longer on the affected than the contralateral side (*P* < 0.0001). The overall specificity and sensitivity were 99 and 51%. Our findings indicate that MIR testing, showing an almost absolute specificity, reliably demonstrates nerve damage beyond doubt, whereas the relatively low sensitivity makes the finding of a normal MIR by no means sufficient to exclude nerve damage. Probably, the dysfunction of a small number of nerve fibres, insufficient to produce a MIR abnormality, may still engender important sensory disturbances. We propose that MIR testing, when used for legal purposes, be considered reliable in one direction only, i.e. abnormality does prove nerve damage, normality does not disprove it.

## Introduction

The inferior alveolar (IAN) and lingual nerves can be injured by many dental or maxillofacial surgical procedures involving the mandible (third molar extraction, placement of endosseous implants, excision, osteotomy) [1]. Due to compression, stretching, or laceration of the alveolar or lingual nerves during surgical steps, some patients complain of sensory disturbances such as pain, paresthesia, dysesthesia and hypoesthesia. These sensory disturbances, which may involve the chin, lower lip, gums, and tongue, are unpleasant conditions that often cause litigation [2].

The masseter inhibitory reflex (MIR), also called “exteroceptive suppression”, is the most used neurophysiological tool for investigating function of the third trigeminal division and mandibular nerves [3]. It consists of a reflex inhibition of the jaw-closing muscles elicited by peri- or intraoral electrical stimulations. MIR comprises an early and a late phase of suppression in the ipsilateral and contralateral masseter muscles. These silent periods are mediated by non-nociceptive A-beta afferents [4] through oligosynaptic (SP1) and polysynaptic (SP2) circuits in the brainstem [5].

Our aim was to assess the MIR sensitivity and specificity in patients with iatrogenic damage to the IAN or lingual nerves, and thus to understand to what extent MIR testing may be used for legal purposes.

## Materials and methods

In a blinded, controlled study, we recorded the MIR in 160 consecutive patients (49 F, 111 M; mean age 44.4 ± 13.5 years) who underwent dental or surgical procedures and thereafter reported sensory disturbances in the territory of the mandibular (IAN or lingual) nerves (third molar extraction: 81; dental implants: 37; mandibular surgery: 22; multiple procedures: 20). All patients were clinically stabilised; the time elapsing between injury and our examination ranged between 2 months and 9 years. Diagnosis of iatrogenic lesion to the mandibular nerves was based on clinical history and examination. Two physicians independently assessed the patients. Only patients with a concordant diagnosis of mandibular nerve lesion were included in the study. Exclusion criteria were neurological diseases other than mandibular nerve lesions. Patients were clinically examined for negative (tactile, pinprick, and thermal hypoesthesia) and positive symptoms (pain, dysesthesias, mechanical allodynia, and pinprick hyperalgesia). All participants gave their informed consent to the procedure.

**Fig. 1:**
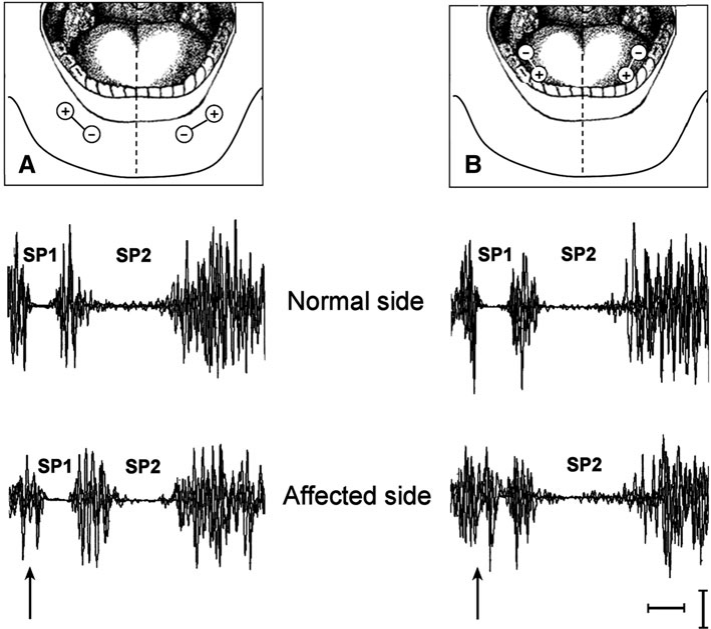
Masseter inhibitory reflex (MIR) in patients with iatrogenic damage to the mandibular nerves. *Left column* inferior alveolar dental nerve testing. *Right column* lingual nerve testing. *Top* schematic drawings showing cathode (−) and anode (+) electrodes position for stimulation of the mental nerve (**a**) and tongue (**b**). *Mid* *and bottom traces* early (*SP1*) and late (*SP2*) components of the MIR after contralateral and affected side stimulations in two representative subjects. Eight trials were superimposed. Calibration 20 ms/200 μV. *Arrows* indicate normal latency. Note that on the affected side the SP1 response is slightly delayed after mental stimulation and it is almost completely absent after tongue stimulation

The MIR was recorded according to the Recommendations of the International Federation of Clinical Neurophysiology (IFCN) [3]. Briefly, subjects were instructed to clench their teeth at maximum strength with the aid of auditory feedback. EMG signals were recorded through surface electrodes from the masseter muscles bilaterally (active electrode over the lower third of the muscle belly and reference electrode about 2 cm below the angle of the mandible). The mental nerve was stimulated transcutaneously with the cathode over the mental foramen and the anode 1 cm laterally. The lingual nerve was stimulated through two adhesive surface electrodes attached 1 cm on the lateral margin of the tongue (Fig. 1). While the subjects were biting in the intercuspal position an electrical square-wave pulse (0.1 ms) was delivered. Stimulus intensity was adjusted to 2.5 times the reflex threshold on the unaffected side (15–45 mA) and kept equal on both sides. In each condition (mental and lingual stimulations), eight trials per side were recorded. Signals were stored for off-line analysis. Two of the authors, blind to the side of damage, took the measurements.

To evaluate the diagnostic accuracy of MIR, we chose to measure only the early SP1 component. The short-latency response SP1 is far more accurate than the long-latency response SP2, because it is supplied by fewer reflex afferents. Additionally, due to the higher number of synapses in the reflex circuit, the late SP2 component is comparatively unstable and is strongly modulated by suprasegmental influences, including psychological factors [3, 5–7].

Because SP1 data had a normal distribution but unequal variances, we analyzed the latency difference between normal and affected side with the *t* test with Welch’s correction. The reflex was considered abnormal when absent or when the SP1 latency difference between normal and affected side was greater than 1.2 ms [3]. To calculate sensitivity and specificity, we used the Fisher’s exact test. Possible associations between pain and reflex abnormality and between pain and sensory deficits were evaluated by Chi square test. Spearman’s *R* correlation coefficient was used to assess correlation between the delay since injury (months) and the severity of nerve damage (latency difference between affected and contralateral side). All results are reported as mean ± SD.

## Results

The patients had damage to the IAN (*n* = 109), the lingual (*n* = 35), or both nerves (*n* = 16).

On the unaffected side, the reflex latency after stimulation of the mental nerve was within the normal range found in 100 healthy subjects in the same laboratory [3] and similar to normal values reported in the literature [6–9]. The mean latency was longer after lingual than mental stimulation (Table 1). Both after mental and lingual stimulation, the latency was significantly longer on the affected than the normal side (*P* < 0.0001) (Fig. 1; Table 1).

**Table 1:** Latency of the masseter inhibitory reflex (mean ± SD) in 160 patients with iatrogenic damage to the mandibular nerves

Stimulation site	Affected	Contralateral	P*
Inferior alveolar nerve (*n* = 125)	12.0 ± 1.3	11.3 ± 0.8	<0.0001
Lingual nerve (*n* = 51)	14.3 ± 2.6	12.9 ± 1.7	<0.0001

P* *t* test with Welch’s correction

Concerning the overall diagnostic accuracy, the MIR was abnormal in 90 nerves and normal in 86 on the affected side whereas on the contralateral side it was abnormal in 2 and normal in 174, which resulted in a strong association (*P* < 0.0001; Fisher’s exact test), with 51% sensitivity (CI: 0.435–0.587) and 99% specificity (CI: 0.959–0.999).

We did not find a significant correlation between the delay since injury and the severity of nerve damage as assessed by the side asymmetry between affected and contralateral side (*P* > 0.10; *R* = 0.1483; Spearman’s correlation coefficient).

Forty-nine patients had neuropathic pains (ongoing or evoked pain). There was no significant association between pain and reflex abnormalities or between pain and sensory deficits (*P* > 0.10).

## Discussion

Our study in a large cohort of patients now shows the diagnostic accuracy of the MIR, a standard neurophysiological tool, in demonstrating iatrogenic damage to the mandibular nerves. We found that the MIR had 99% specificity and 51% sensitivity, i.e. MIR testing reliably demonstrates nerve damage beyond doubt, whereas the finding of a normal MIR is by no means sufficient to exclude nerve damage. Probably the dysfunction of a small number of nerve fibres, insufficient to produce a MIR abnormality, may still engender important sensory disturbances.

We propose that MIR testing, when used for legal purposes, be considered reliable in one direction only, i.e. abnormality does prove nerve damage, normality does not disprove it.

Although it would be reasonable to expect some degree of recovery with time, because of spontaneous healing and reinnervation, we found no correlation between the delay since injury and MIR abnormality. This is probably due to the characteristics of our patient sample: all patients were examined when at least 2 months had elapsed from injury (i.e. when no further healing is expected to occur) and 146 out of 160 were examined beyond 4 months (i.e. when, given the short length of the lingual and inferior dental nerves, the process of reinnervation was completed). Given the importance of this aspect, however, we are now planning a study in the acute phase after injury.

Other neurophysiological tests may also assess mandibular nerve function: trigeminal-evoked potentials, nerve conduction study, and blink reflex. Although some authors used trigeminal somatosensory-evoked potentials to study IAN injuries after surgical procedures, the reliability of these signals has been questioned because, rather than reflecting genuine brain activity, probably they result from volume-conducted muscle signals, as they disappear in the curarized subject [10, 11]. Consistently, the IFCN recently recommended to investigate the trigeminal function with reflex rather than evoked potential studies [12]. The IAN nerve conduction study is a reliable method, but it is undeniably invasive (the recording needle-electrode is inserted below the zygomatic arc to a depth of about 4.5 cm) [13]. Unlike the above techniques, the blink reflex after mental or lingual stimulation seems a promising alternative to the MIR. It has been widely studied in patients with orofacial pains or trigeminal neuropathy and, in combination with the IAN conduction study, showed sensitivity (59%) and specificity (100%) values very similar to those we found in our patients. The patients with iatrogenic damage after dental procedures, however, still represent a comparatively small sample [13, 14]. Damage to the lingual nerve after third molar extraction can also be tested with an interesting method, very similar to ours (even though the main measure is not the reflex latency) [15]. Also in this case, the number of patients is still too small to test diagnostic accuracy.

Whereas most patients had hypoesthesia, less than one-third had pain, probably because mechanical injury mainly damages large myelinated, non-nociceptive fibres, and tends to spare small nociceptive fibres. Accordingly, because MIR is mediated by non-nociceptive Aβ fibres [4], and it does not provide any information on nociceptive pathways, we found that MIR abnormalities were unrelated to pain. Alternatively, the complexity of pain mechanisms at orofacial level simply makes it unreasonable to expect a direct correlation between pain and number of damaged primary afferents [16]. In particular, we cannot exclude that plastic changes in the central nervous system contributed to the sensory disturbances reported by our patients. According to this hypothesis, sensory disturbances could be triggered by the nerve damage and persist after nerve recovery due to central mechanisms such as central sensitization.
